# Multi-drug resistant extended-spectrum beta-lactamase producing *E. coli* and *Salmonella* on raw vegetable salads served at hotels and restaurants in Bharatpur, Nepal

**DOI:** 10.1186/s13104-019-4557-9

**Published:** 2019-08-16

**Authors:** Sanjeep Sapkota, Sanjib Adhikari, Asmita Pandey, Sujan Khadka, Madhuri Adhikari, Hemraj Kandel, Sandhya Pathak, Asmita Pandey

**Affiliations:** 1 Department of Microbiology, Birendra Multiple Campus, Tribhuvan University, Bharatpur, Nepal; 2 Key Laboratory of Protein and Peptide Drugs, Institute of Biophysics, Chinese Academy of Sciences, Beijing, China; 3 Department of Life Sciences, Biochemistry and Molecular Biology, Central China Normal University, Wuhan, China

**Keywords:** Salads, *E. coli* O157: H7, Antibiograms, Multi-drug resistant, ESBL, Hotels and restaurants

## Abstract

**Objective:**

Antimicrobial resistance among the bacteria present in ready-to-eat foods like vegetable salads is an emerging concern today. The current study was undertaken to investigate the presence of multi-drug resistant extended-spectrum β-lactamase (ESBL) producing *E. coli* and *Salmonella* spp. in raw vegetable salads served at hotels and restaurants in Bharatpur. A total of 216 salad samples were collected from three different grades of hotels and restaurants and examined for the presence of *E. coli* and *Salmonella* spp. in Microbiology laboratory of Birendra Multiple Campus by conventional microbiological techniques.

**Results:**

Out of 216 samples, 66 samples (35.2%) showed the presence of *Salmonella* spp. whereas *E. coli* was recovered from 29 (13.4%) samples of which 3 samples harbored *E. coli* O157: H7. Antibiotic susceptibility testing revealed that 9 (13.6%) *Salmonella* and 4 (13.8%) *E. coli* isolates were detected as multi-drug resistant. Total ESBL producers reported were 5 (7.57%) *Salmonella* and 4 (13.8%) *E. coli*. The study also assessed a significant association between occurrence of *E. coli* and *Salmonella* with different grades of hotels and restaurants, personal hygiene and literacy rate of chefs and with the type of cleaning materials used to wash knives and chopping boards (p < 0.05). The findings suggest an immediate need of attention by the concerned authorities to prevent the emergence and transmission of food-borne pathogens and infections antimicrobial resistance among them.

## Introduction

Salad is any food preparations made up of a mixture of chopped or sliced vegetables that provides a good source of minerals and dietary fiber of low fat and calories to the consumer [1]. Salad vegetables are consumed without any form of heat treatment, sometimes without washing, thus, has the possibility of causing food-borne diseases [2]. Some previous studies have revealed the presence of *E. coli, E. coli* O157: H7, *Salmonella* spp., *Enterobacter aerogenes*, *Pseudomonas* spp., *Klebsiella* spp., *Providencia* spp., *Listeria monocytogenes* and *Proteus* spp. from vegetable salads [3–6].

Drug resistance is one of nature’s not ever ending processes which may be due to a pre-existing factor in the organisms or results from the acquired factors [7]. The enteric bacteria in fecal flora are often stated to be highly resistant and *E. coli* is reported to be the main transporter of antimicrobial resistance [8]. Beta-lactamase production is the most important mechanism of resistance to penicillins and cephalosporins [9]. Bacterial species that hold extended-spectrum beta-lactamase (ESBL) genes reside in the gastrointestinal tract and food is the key route of them [10]. To the best of our knowledge, no any study has yet been made in Nepal that endeavors to examine the bacterial quality of green salads. Hence this study was carried out to screen multi-drug resistant extended spectrum beta-lactamase producing *E. coli* and *Salmonella* on raw vegetable salads served at hotels and restaurants in Bharatpur.

## Main text

### Study design and setting

A descriptive cross-sectional study was carried out for 5 months from November 2017 to March 2018 in Bharatpur metropolitan city. The salads samples were collected using random sampling methods without repetition from different grades of hotels and restaurants in the city. The sample size was determined in accordance with the prevalence rate based on the previous study [4]. A total of 216 salad samples were included in this study.

### Methodology

A total of 216 ready-to-eat salad samples (72 radish, 72 carrots and 72 cucumbers) were collected in sterile zip-locked plastic bags from three different grades of hotels and restaurants in Bharatpur city and transported aseptically to the Microbiology laboratory of Birendra Multiple Campus within 1 h for further analysis. The hotels and restaurants were categorized into three grades: A, B and C based on the cost, economic class of the customers and number of staffs involved in serving [11]. Twenty-five gram of salad sample was mixed with 225 ml sterile water and left covered for 30 min. Then, 1 ml of the sample was inoculated in buffered peptone water and Selenite F broth and incubated at 37 °C for 24 h. One loopful of the culture was streaked on Eosine Methylene Blue agar and Xylose Lysine Deoxycholate agar for the identification of *E. coli* and *Salmonella* spp. respectively and incubated at 37 °C for 24 h. The suspected colonies after incubation were sub-cultured on Nutrient agar. Identification of bacterial isolates was carried out based on their cultural, morphological and biochemical characteristics [12]. For the preliminary identification of *E. coli* O157: H7, *E. coli* isolates were streaked on the MacConkey Sorbitol agar and incubated at 37 °C for 24 h and colorless colonies were suspected as *E. coli* O157: H7. Later, the slide agglutination test using anti-O157 and flagellar H7 serum was done (Defico, USA) to confirm *E. coli* 0157:H7 strains.

Antibiotic susceptibility tests (AST) were performed by modified Kirby Bauer’s disc-diffusion method [13]. Antimicrobial discs amoxicillin (10 µg), cotrimoxazole (25 µg), ciprofloxacin (5 µg), gentamicin (10 µg) and azithromycin (15 µg) for *Salmonella* and in case of *E. coli*, gentamicin (10 µg), ciprofloxacin (5 µg), cotrimoxazole (25 µg), ampicillin (10 µg) and chloramphenicol (30 µg) were used in the study. Resistance shown to at least three or more antibiotics of different structural classes was considered MDR as described elsewhere [14, 15]. Screening of ESBL was performed by using ceftazidime (30 µg) and cefotaxime (30 µg) discs. The zone of inhibition (ZOI) ≤ 22 mm for ceftazidime and ≤ 27 mm for cefotaxime was considered as potential ESBL producer as recommended by Clinical and Laboratory Standards Institute (CLSI). Possible ESBL producers were subjected to combined disc test for phenotypic confirmation as recommended by CLSI [16]. The combination of ceftazidime and cefotaxime alone and in combination with clavulanic acid (CA) (10 µg) were used for the confirmation of ESBL producing isolates. An increase ZOI of ≥ 5 mm for either antimicrobial agent tested in combination with CA versus its zone when tested alone was interpreted as positive for ESBL production. *E. coli* ATCC 25922 and *Salmonella* Typhimurium ATCC 14028 were used as reference strains for quality control.

Raw data obtained in this study were tabulated in SPSS V.20 and Chi square test was performed. p ≤ 0.05 was assigned as significant.

### Results

#### Distribution of *E. coli* and *Salmonella* in salads

A total number of 216 salad samples were collected and analyzed for the presence of *Salmonella* and *E. coli*. *Salmonella* was isolated from 66 (30.56%) samples while *E. coli* was isolated from 29 (13.43%) samples, out of which 3 (10.34%) were found to be *E. coli* O157: H7.

#### Association of various variables with the rate of bacterial contamination

A total of 216 salad samples were analyzed for the presence of *E. coli* and *Salmonella*. Salads served in Grade C restaurants showed higher contamination of *E. coli* (20.83%) and *Salmonella* (41.64%) as compared to Grade A and B types (p ≤ 0.05). Similarly, salads prepared by the illiterate chefs were found more prone to contamination by *E. coli* (44%) and *Salmonella* (66.7%) (p ≤ 0.05). The high incidence of contamination by *E. coli* (16.67%) and *Salmonella* (32.8%) were observed in the salads prepared by the non-gloved chefs (p ≤ 0.05). Chopping boards and knives washed by water showed higher contaminations of *Salmonella* (44.4%) in comparison to those washed by detergents and soaps (p ≤ 0.05) (Table 1).

**Table 1:** Association of various variables with the rate of bacterial contamination

S. no.	Attributes	Sample from	No. of sample	Isolates			
				*E. coli*		*Salmonella*	
				Contaminated sample	p-value	Contaminated sample	p-value
1	Grades	Grade A	72	4 (5.55%)		14 (19.44%)	
		Grade B	72	10 (13.88%)	0.015*	22 (30.55%)	0.015*
		Grade C	72	15 (20.83%)		30 (41.64%)	
2	Literacy	Literate	207	25 (12%)	0.021*	60 (29%)	0.025*
		Illiterate	9	4 (44%)		6 (66.7%)	
3	Personal hygiene	Gloved	54	2 (3.7%)	0.016*	1 (5.6%)	0.015*
		Non-gloved	162	27 (16.67%)		65 (32.8%)	
4	Cleaning materials	Soap/detergent	171	19 (11%)	0.052	46 (26.9%)	0.023*
		Water	45	10 (22.2%)		20 (44.4%)	

* Significant at 5% level of significance

#### Antibiotic susceptibility pattern

AST was also performed for all the *E. coli* and *Salmonella* isolates against different antibiotics like ampicillin, cotrimoxazole, chloramphenicol, ciprofloxacin, azithromycin, amoxicillin and gentamicin. More than half of the *E. coli* isolates (58.60%) were found to be resistant to ampicillin. Gentamicin was the most effective antibiotic as 96.60% of the isolates were found to be sensitive to it followed by cotrimoxazole killing 93.20% of *E. coli* isolates. The least effective antibiotic was ampicillin which inhibited the growth of 41.40% of *E. coli* isolates. Out of 29 isolates of *E. coli*, 4 (13.80%) were MDR. Likewise, all *Salmonella* isolates were found to be resistant to amoxicillin. Cotrimoxazole was the most effective antibiotics as 97% of *Salmonella* isolates were found to be sensitive to it followed by gentamicin killing 95.5% of *Salmonella* isolates. The least effective antibiotics was ciprofloxacin which inhibited the growth of only 4% *Salmonella* isolates followed by amoxicillin to which all the isolates were resistant. Out of 66 isolates of *Salmonella* subjected to AST, 19 (28.78%) were MDR (Table 2).

**Table 2:** Antibiotic susceptibility pattern of isolates

SN	Antibiotics	Susceptibility pattern					
		Susceptibility pattern of *Salmonella*			Susceptibility pattern of *E. coli*		
		S	I	R	S	I	R
1	Azithromycin	77.30%	0%	22.70%	NT	NT	NT
2	Amoxicillin	0%	0%	100%	NT	NT	NT
3	Ciprofloxacin	6%	80.30%	13.70%	82.80%	0%	17.20%
4	Cotrimoxazole	97%	0%	3%	93.20%	3.40%	3.40%
5	Gentamicin	95.50%	0%	4.50%	96.60%	3.40%	0%
6	Chloramphenicol	NT	NT	NT	62.10%	24.10%	13.80%
7	Ampicillin	NT	NT	NT	41.40%	0%	59.60%

*S* sensitive, *R* resistant, *I* intermediate, *NT* not tested

#### Frequency of ESBL producing *E. coli* and *Salmonella* isolates

Out of 95 isolates, 12 isolates gave ESBL screening test positive. Four isolates of *E. coli* and 5 isolates of *Salmonella* accounting a total of 9 (9.47%) were further confirmed as ESBL producers (Table 3).

**Table 3:** ESBL detection by combination disk-method

Organism	Significant growth	Screening test positive	Confirmatory test positive (combination disk method)
*E. coli*	29	5	4 (13.8%)
*Salmonella*	66	7	5 (7.57%)
Total	95	12	9 (9.47%)

### Discussion

A total of 216 salad samples were analyzed in the present study, out of which 13.45% of samples were contaminated by *E. coli*. A similar study was performed in Kashan city of Iran which reported *E. coli* in 85% salad samples [17]. In a study from India, *E. coli* was found in 38.3% of different vegetable salads [18]. These two studies show a higher level of contamination by *E. coli* than our study. Similarly, the prevalence of *Salmonella* reported in this study was 30.6%. It is lower than the study done in India, which reported only 3.33% of vegetables salads contaminated with *Salmonella* spp. [19]. A similar study was conducted in Nigeria which reported the presence of 25.0% salad samples contaminated by *Salmonella* [20]. A higher incidence *Salmonella* might be associated with the increased thermal resistance of *Salmonella* in chicken litter applied in the field [21]. In this study, *E. coli* O157: H7 have been reported in 1.4% salad samples. A similar study reported *E. coli* O157: H7 in 1.3% (6/480) of the raw salad vegetables [22]. Vegetables grown in soil fertilized by animal manure have a greater chance to be contaminated with *E. coli* O157: H7 [23].

A higher number of salad samples harboring *Salmonella* (41.64%) and *E. coli* (20.83%) were isolated from the Grade C restaurants compared to Grade A and B in the current study (p ≤ 0.05). This might be due to the fact that chefs were unaware of health as well as personal hygiene, and water used for washing salad samples might not be disinfected properly in Grade C types. Distribution of *E. coli* and *Salmonella* also vary based on the literacy rate of the chefs [24]. Our study revealed a higher incidence of *E. coli* (44%) and *Salmonella* (66.7%) in salads made by illiterate chefs (p ≤ 0.05). Illiterate chefs might lack the knowledge of consumer’s health and they might cross-contaminate the salads in the course of preparation. Personal hygiene of the chefs may aid in the variation in the distribution of *Salmonella* isolates [25]. Salads prepared by the non-gloved chefs showed a high rate of contamination—*Salmonella* (32.8%) and *E. coli* (16.67%) (p ≤ 0.05). This finding is in tune with a work which revealed that non-gloved vendors served highly contaminated ready to eat foods than gloved vendors (p < 0.01) [26]. Contaminated hands can easily transmit the pathogens like *Salmonella* and *E. coli* O157H7 in foods [27]. Obviously, non-gloved chefs might carry dirt and dust particles which include the higher number of microbes that can contaminate the salads while preparing. Wide fluctuation in the prevalence of *Salmonella* in salads can be observed in the cleaning trend of chopping board and knife [28]. More isolates of *Salmonella* (44.4%) was found in salads cut in the chopping boards and knives which are regularly washed by soaps and detergents (p ≤ 0.05). A study showed a significant coliform reduction (p ≤ 0.05) for all the methods of disinfection. Disinfection processes can actively reduce the bacterial loads from inanimate surfaces, so a significant coliform reduction can be achieved [29, 30].

On microbial testing, cotrimoxazole and gentamicin were the most effective antibiotics for both *E. coli* and *Salmonella* isolates. Amoxicillin was the most ineffective antibiotic as 100% of the *Salmonella* isolates were found to be resistant to it. A similar study conducted in Nigeria found all the *Salmonella* isolates from salads were resistant to amoxicillin [20]. This could due to the easy hydrolysis of the β-lactam ring by *Salmonella* as well as the frequent usage of the drug due to its low cost leading to the development of resistance by most bacteria.

In this study, 9 (13.6%) *Salmonella* isolates and 4 (13.8%) *E. coli* were found to be MDR. The prevalence of MDR in *Salmonella* and *E. coli* has been reported comparatively higher in some other studies. A study in major markets in Chittagong of Bangladesh reported that all the *Salmonella* and *E. coli* isolates from green salads were MDR [31]. Moreover, another similar work conducted in the same city, Chittagong, found that 48.2% of isolates were MDR [11]. The problem of antibiotics resistance has been increasing day by day and it has become a serious burden in the medical world. The frequency of ESBL producers for *Salmonella* was 5 (7.57%) and *E. coli* was 4 (13.8%) in the present study. A study conducted by Raphael et al. observed an ESBL incidence rate of 2.3% among bacterial isolates from spinach [32]. A similar study was conducted by Bezanson et al. who detected 1.9% of ESBL producers from lettuce [33]. These two studies revealed a low prevalence of ESBL producers than our work. Over-consumption and haphazard use of beta-lactam antibiotics might have contributed to the emergence of ESBL producers.

### Conclusions

The results of this study reveal that the safety aspect of fresh vegetable salads served at the hotels and restaurants in Bharatpur city is unacceptable from microbiological point of view. Poor personal hygiene of the chefs, improper handling and lack of knowledge on consumer health are some of the factors the concerned authorities should pay immediate attention to control the transmission of food-borne pathogens and emergence of antimicrobial resistance among them.

## Limitations

The study only determines the prevalence of *E. coli* and *Salmonella* species and their MDR pattern including ESBL production. The source of contamination of these items was not assessed however. The present study also lacks the molecular characterization of the isolates. Other isolates besides *E. coli* and *Salmonella* were not included in the study. Food safety knowledge of the chefs was not assessed. Similarly, the bacterial quality of water used for the preparation of salads was not tested.

## Data Availability

All the data obtained during this study are available within the article.

## Abbreviations

ESBL: extended spectrum beta-lactamase
MDR: multi-drug resistant
DIZ: diameter of the inhibition zone
RDA: recommended daily allowance
MHA: mueller hinton agar
AST: antimicrobial susceptibility testing
CLSI: Clinical and Laboratory Standards Institute
